# Design of Polymeric and Biocompatible Delivery Systems by Dissolving Mesoporous Silica Templates

**DOI:** 10.3390/ijms21249573

**Published:** 2020-12-16

**Authors:** Ana Rodríguez-Ramos, Laura Marín-Caba, Nerea Iturrioz-Rodríguez, Esperanza Padín-González, Lorena García-Hevia, Teresa Mêna Oliveira, Miguel A. Corea-Duarte, Mónica L. Fanarraga

**Affiliations:** 1 Grupo de Nanomedicina, Instituto Valdecilla-IDIVAL, Herrera Oria s/n, 39011 Santander, Spain; arodriguez@idival.org (A.R.-R.); nerea.iturrioz@alumnos.unican.es (N.I.-R.); esperanza.padin@alumnos.unican.es (E.P.-G.); monica.lopez@unican.es (M.L.F.); 2 CINBIO, Universidade de Vigo, 36310 Vigo, Spain; lmarincaba@gmail.com (L.M.-C.); teresa.mena.oliveria@gmail.com (T.M.O.); macorrea@uvigo.es (M.A.C.-D.); 3 Southern Galicia Institute of Health Research (IISGS), CIBERSAM, 36312 Vigo, Spain; 4 Molecular Biology Department, University of Cantabria, 39011 Santander, Spain

**Keywords:** silica particle, dissolution, nanocarrier system, delivery, polymer

## Abstract

There are many nanoencapsulation systems available today. Among all these, mesoporous silica particles (MSPs) have received great attention in the last few years. Their large surface-to-volume ratio, biocompatibility, and versatility allow the encapsulation of a wide variety of drugs inside their pores. However, their chemical instability in biological fluids is a handicap to program the precise release of the therapeutic compounds. Taking advantage of the dissolving capacity of silica, in this study, we generate hollow capsules using MSPs as transitory sacrificial templates. We show how, upon MSP coating with different polyelectrolytes or proteins, fully customized hollow shells can be produced. These capsules are biocompatible, flexible, and biodegradable, and can be decorated with nanoparticles or carbon nanotubes to endow the systems with supplementary intrinsic properties. We also fill the capsules with a fluorescent dye to demonstrate intracellular compound release. Finally, we document how fluorescent polymeric capsules are engulfed by cells, releasing their encapsulated agent during the first 96 h. In summary, here, we describe how to assemble a highly versatile encapsulation structure based on silica mesoporous cores that are completely removed from the final polymeric capsule system. These drug encapsulation systems are highly customizable and have great versatility as they can be made using silica cores of different sizes and multiple coatings. This provides capsules with unique programmable attributes that are fully customizable according to the specific needs of each disease or target tissue for the development of nanocarriers in personalized medicine.

## 1. Introduction

Many drugs of extraordinary pharmacological interest have been discarded because of their untargeted toxicity or premature degradation. Typical examples are some anti-cancer drugs or antibiotics that, to reach the sites of action at the right concentrations to obtain the desired effects, cause significant damage to healthy tissues. Therapeutic compound encapsulation in nanocarriers is an excellent alternative to traditional formulations. It can help poorly soluble or labile therapeutic compounds to achieve potent therapeutic effects at their target site, minimizing the drug’s side effects and toxicity issues, while significantly improving the drug’s biodistribution or stability.

There are many possibilities in drug delivery, each nanosystem with its relative advantages and disadvantages. Different types of nanomaterials have been used as nanocarriers. The most employed are the following: liposomes [1,2], dendrimers [3,4,5], polymeric micelles [6,7], inorganic particles [8], carbon nanotubes [9], or silica-based materials [10,11,12]. Mesoporous silica particles (MSPs) have attracted much attention thanks to their singular properties: they are biocompatible; safe; and display a versatile, customizable, and controllable nature [13,14]. These nanomaterials exhibit modifiable surface properties, a huge surface-to-weight ratio (700–1000 m^2^/g) [15,16] with a well-defined internal mesopore structure (from 2 to 10 nm of diameter), and a large pore volume (0.6–1 cm^3^/g) [17]; thus, they can be adapted to carry many different bioactive molecules.

MSPs can display size ranges from the nano-scale (50 nm) up to the submicron-scale (500 nm) and their shape and surface can be custom-designed, thus allowing many different possibilities to carry different drugs [14,15,16,18,19,20,21]. These nanoparticles have been loaded with molecules of various sizes and natures, such as drugs, nucleic acids, or proteins, and can be synthesized containing different nanomaterials either in the core or the surface (for example, silica particles can be synthesized containing magnetic or fluorescent nanoparticles). Most therapeutic compounds can be loaded inside their mesopores through a simple diffusion mechanism. Upon particle loading, these are clogged using different coating systems or gatekeepers to temporarily limit drug release until the carrier reaches its final destination. Ideally, the MSP capsule should dissolve at the target tissue, producing a drug burst release effect.

However, MSPs also have a downside. Many studies demonstrate the dissolution of silica upon exposure to different biological conditions depending on factors such as temperature, pH, surface modifications, or the chemical composition of the medium surrounding the particle [22,23,24,25,26,27,28,29,30]. Indeed, MSPs have been claimed to be rapidly degraded in physiological media [25]. It was observed that silica dissolution rates are higher in lung fluid, followed by body fluid (similar to phosphate-buffered saline (PBS)), although they were more stable in gastric juice [31]. Similarly, at the subcellular level, MSPs are rapidly dissolved in the culture media or intracellularly, compared with the lysosome [32]. In general, silica particles dissolve faster in media of physiological pH containing amino-rich compounds such as cell culture medium, although serum seems to reduce degradation [22,30]. These facts make MSPs carrier vehicles unreliable from a therapeutic point of view because drug release is not controllable, which is a clear drawback for the applications of plain MSPs as drug nanocarriers.

One of the most commonly used methods to control therapeutic compound release from MSPs consists of the polyelectrolyte (PEE) multilayer coating the surface of the particles [10,33,34,35]. This method, prepared following the layer-by-layer (LbL) technique, allows full customization of MSPs, for instance, with many different types of PEE (i.e., fluorescent PEE, cationic, anionic), or including, among many others, nanoparticles, carbon nanotubes, or polypeptidic ligands in the coating. Moreover, the resistance to rupture of the coating can be modified by changing the nature and number of PEE layers.

Taking into account all these issues, we decided to use MSPs as transitory templates that are eventually removed (herein named sacrificial) by the dissolution of the SiO_2_ to create polymeric capsules assembled with biocompatible polymers of different natures and functionalities. This procedure allows significantly reducing the inorganic compound (Si) of the final nanocarrier, thus resulting in fully biocompatible and biodegradable squeezable capsules.

## 2. Results

### 2.1. Optimization of the MSP Dissolution Process

**Figure 1:**
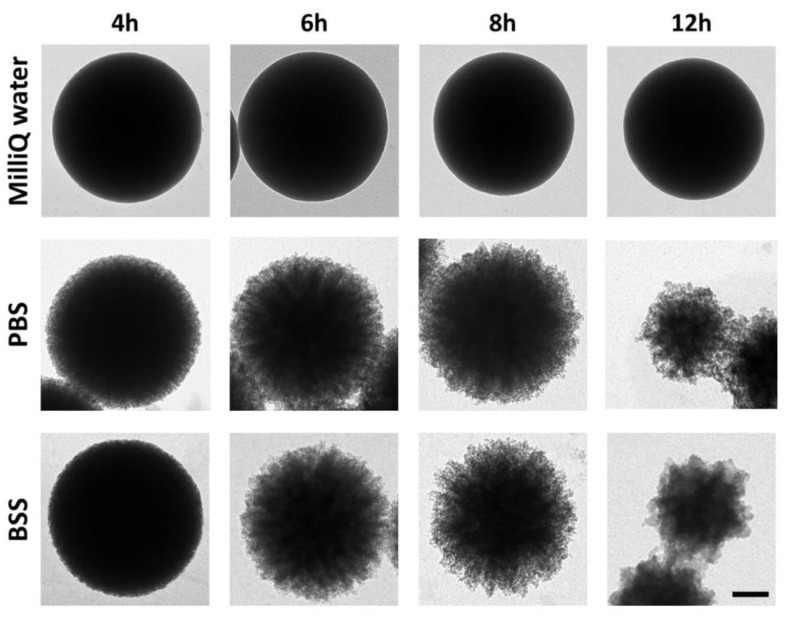
Degradation of mesoporous silica particles (MSPs) in different media. Representative transmission electron microscopy (TEM) images of bare MSPs after incubation at 37 °C for 4, 6, 8, and 12 h in MilliQ water, phosphate-buffered saline (PBS), or a balanced salt solution (BSS). All images are taken at the same magnification. The scale bar is 100 nm.

To optimize the dissolution process, MSPs’ cores were incubated with different solutions at 37 °C and different times. Figure 1 shows TEM images of MSPs incubated in PBS, BSS, and MilliQ water. Both PBS and BSS are typical isotonic buffered (pH 7.4) solutions commonly used in cellular nanocarrier experiments. MilliQ water (pH 5.5) was found to preserve the structure of the MSPs better than any of the two saline solutions containing several inorganic ions—which include potassium, sodium, or calcium (see Materials, Section 2.5)—which could assist the dissolution of silica. Incubation of the MSPs in both saline solutions produced a significant degradation of the surface of the particles after a few hours. Degradation was more noticeable at the edge of the particles. Partial particle crumbling was evident after 6–8 h.

Previous literature and studies [23,26,32,36,37] support the conclusion that MSPs are dissolved in these media. Indeed, silica disappearance is concomitant with the progressive detection of Si species in the media [32]. Hence, we believe these experiments reveal that isotonic solutions (ca. 150 mM salt) at a physiological pH (7.4) significantly accelerate MSPs’ decomposition. However, the reaction presents many variables. Silica dissolution is affected by the intrinsic properties of the particle [28,38]. In particular, we observed (i) the size of the particle, (ii) the size of the mesopores, and (iii) the silica density. However, dissolution is also affected by (i) the incubation time; (ii) the concentration of particles in the dissolution medium; and (iii) the composition of the medium, namely the pH and ionic strength. These experiments served to standardize the conditions for the complete dissolution of the described 500 nm diameter MSPs. In general, we estimated that 100 µg/mL MSPs are completely dissolved in approximately 24 h upon incubation in isotonic buffered saline solutions at 37 °C upon mild rotation.

### 2.2. Producing and Customizing Polymeric Capsules by Dissolving the MSP Core

**Figure 2:**
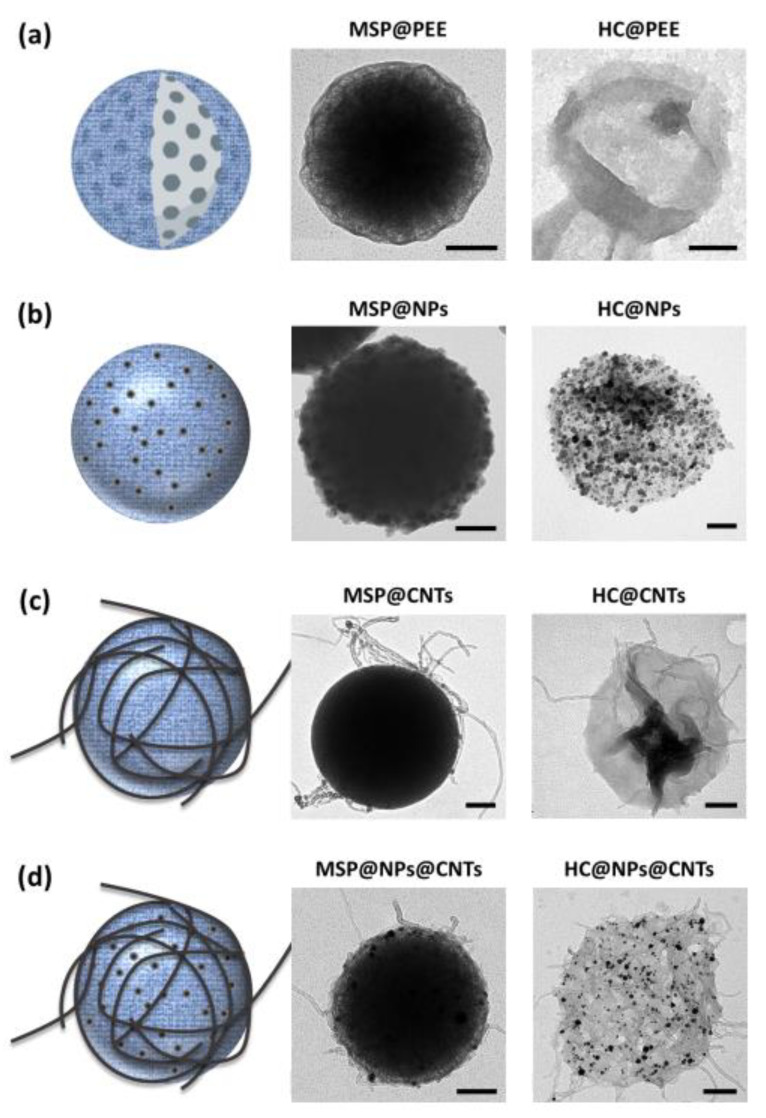
Diagrams and TEM images of different examples of hollow polyelectrolyte (PEE) capsules. MSPs, before and after silica core dissolution. (**a**) MSP coated with PEE and the resulting hollow capsule (HC@PEE). (**b**) PEE capsules decorated with nanoparticles (NPs), in this case, CoFe_2_O_4_ NPs. (**c**) PEE capsules decorated with carbon nanotubes (CNTs), in this case, oxidized multi-walled CNTs (MWCNTs). (**d**) Example of PEE hollow capsules decorated with NPs and CNTs, in this case, Pt NPs and ox-MWCNTs. Scale bar corresponds to 100 nm.

MSPs are often used as templates for layer-by-layer deposition of PEEs. These coatings are commonly used in MSPs’ carriers to prevent the diffusion of the encapsulated compound to the surrounding media. So, we now tested whether, with the PEE coating, the MSP would dissolve to produce flexible hollow capsules using silica sacrificial templates. To do this, we coated MSPs with alternate PEE multilayers (as described in the Methods section), and incubated the coated particles in the saline buffered solution as described. Figure 2a shows TEM images demonstrating how the PEE coating does not significantly interfere with the dissolution of the silica, resulting in hollow PEE capsules using this simple procedure.

In view of these results, we decided to go one step further, customizing the PEE coating by including nanomaterials (i.e., nanoparticles and carbon nanotubes) in the coating. These variations open new ways to endow the capsules with different properties, to be used for resonance imaging (MRI) [39] or hyperthermia, by simply applying an external magnetic field preventing the agglomeration of the nanoparticles [40,41], and could lead to active targeting. In the sample, we included magnetic cobalt ferrite (CoFe_2_O_4_) nanoparticles in the polymeric coating. In particular, these particles were coated by two polymeric PEE bilayers (PDDA + PSS) that included CoFe_2_O_4_ magnetic nanoparticles in between the PEE layers (Materials section). Upon MSPs’ core dissolution, these resulting magnetic capsules (HC@CoFe_2_O_4_) were transparent, flexible structures decorated with nanoparticles (Figure 2b). Similarly, we produced hybrid nanomaterial using oxidized multi-walled nanotubes (CNTs) on PEE (PEM) coated MSPs. CNTs can serve to improve the interaction of the capsule with the cellular surface and to trigger endo-lysosomal escape [42], thus offering new possibilities in drug delivery. Figure 2c shows empty HC@CNTs capsules. In the same way, it is also possible to combine different types of nanomaterials in the same coating resulting in innumerable design possibilities to customize the capsules ad hoc. For instance, in Figure 2d, we show PEE hollow capsules produced Pt NPs and CNTs.

### 2.3. Producing Protein Capsules Using MSP Sacrificial Cores

**Figure 3:**
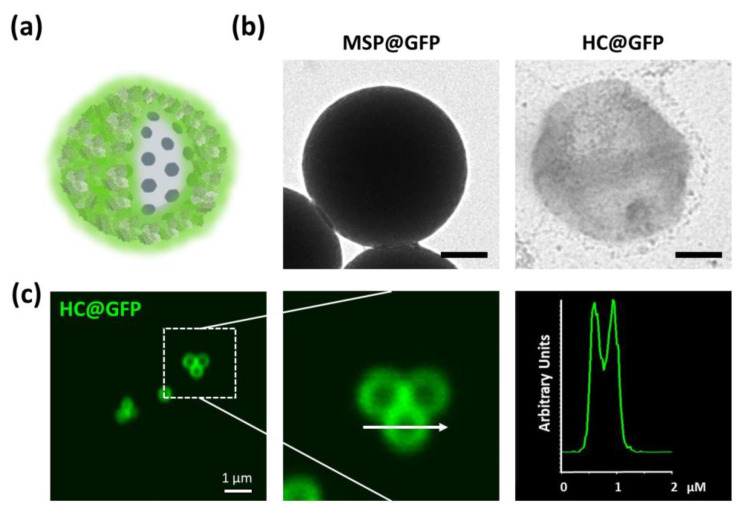
Fluorescent protein nanocapsules. (**a**) Schematic representation of green fluorescence protein (GFP) coated MSPs. (**b**) TEM images of GFP-coated MSPs before (left) and after silica dissolution (right). Resulting HC@GFP capsules after protein crosslinking and MSP core dissolution. (**c**) Confocal microscopy single plane image of the fluorescent HC@GFP nanocapsules. (right) Fluorescent protein profile obtained of one of the capsules (arrow) where a central cavity devoid of protein (empty core) can be identified. Scale bar corresponds to 100 nm.

As a more biocompatible alternative to synthetic polymers, we decided to create hollow capsules using protein, in order to obtain fully biodegradable structures. As proof-of-concept, we used purified green fluorescent protein (GFP) to create HC@GFP capsules. The protein was bioconjugated on the capsules as previously described [43], and was crosslinked using a 0.25% glutaraldehyde solution, a method commonly used in pharmaceutical technology (Figure 3) [44,45]. Similar capsules were produced using bovine serum (FBS) as a sample of alternative protein. Both crosslinked proteins formed a mesh that, as observed to the PEE coating, also allowed MSP dissolution and the formation of hollow capsules. The intrinsic fluorescence of the GFP served to document the shape of the capsules using confocal fluorescent microscopy. An empty core in the center of the structure was identified (Figure 3c).

### 2.4. Loading the Polymeric/Protein Capsules

**Figure 4:**
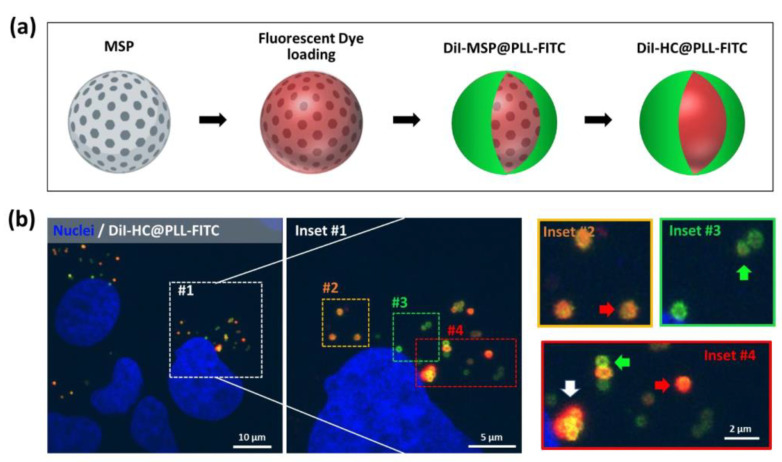
(**a**) Scheme of fluorescent PEE-DiI filled capsule design. MSPs are loaded with DiI and coated with a fluorescent PEE, in this case, fluorescein isothiocyanate (FITC) poly-L lysine (PLL-FITC). (**b**) Single plane confocal images of Hela cells treated with DiI filled PEE capsules (DiI-HC@PLL-FITC) for 96 h. (**inset #1**) Magnification of the area allows the identification of individual intracellular capsules and their contents. (**inset #2**) Filled particles loaded with DiI (orange). (**inset #3**) FITC empty fluorescent capsules (green). (**inset #4**) Capsules releasing DiI (white arrow) intermingle with empty capsules (green arrows) and filled intact capsules (red arrow) within the cellular cytoplasm. Cell nuclei are stained with Hoechst (blue channel).

**Figure 5:**
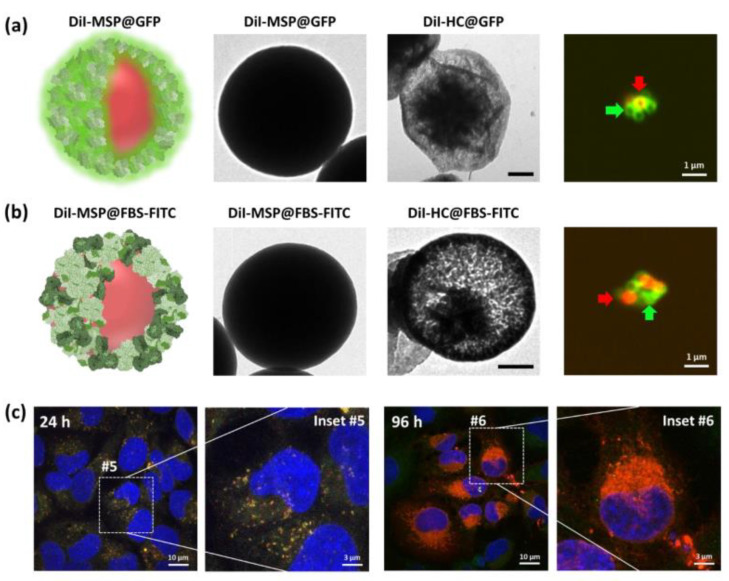
Schematic representation of polymeric protein capsules loaded with DiI. TEM images of DiI-loaded MSP coated with (**a**) purified GFP, or (**b**) FITC-stained serum proteins (FITC-FBS). (right) Single plane confocal images of DiI-HC@GFP and DiI-HC@FBS-FITC. Red arrows point at DiI filled capsules and green arrows to empty shells. (**c**) HeLa cell cultures were treated with DiI-HC@FBS-FITC for 24 (left) and 96 h (right). Internalization of the DiI-MSPs@FBS-FITC capsules is very evident after 24 h. The yellow color of the capsules (yellow = green + red, corresponding to FITC+DiI, respectively) means that systems are still intact inside cells. After 96 h incubation, the red color in the endoplasmic reticulum of most cells suggests dye bust release. Nuclei are stained with Hoechst (blue channel). Insets **#5** and **#6** shown high magnification of individual cells containing the DiI-loaded capsules. Scale bars on TEM images correspond to 100 nm.

Next, we decided to do a proof-of-concept to demonstrate that these polymer capsules could be filled with compound to be used as encapsulating systems for *in cellulo* delivery. For this purpose, we filled the capsules with DiI, a fluorescent lipophilic carbocyanine dye that displays a high photostability and, if released, can be easily documented using confocal microscopy. The DiI was loaded into MSPs by infiltration to produce DiI-MSPs. Filled particles were repeatedly now coated with both, PEE, or proteins (both, FBS-FITC, or GFP) to produce fluorescent DiI-MSP@PLL-FITC, DiI-MSP@FBS-FITC, and DiI-MSP@GFP particles, respectively (Figure 4a and Figure 5a,b). Dissolution of the MSP cores infiltrated with DiI was performed in saline buffered salt solution at 37 °C in mild rotation. As previously observed, silica dissolution occurred at a similar rate for both, PEE, and protein capsules after 24 h of incubation. This resulted in flexible, polymeric, and fluorescent DiI filled capsules (Figure 5a,b).

### 2.5. Polymeric Capsules are Engulfed by HeLa Cells and Deliver Their Contents

To test if DiI filled capsules were capable of delivering compounds intracellularly, we added both, polymeric DiI-HC@PLL-FITC, and protein DiI-MSP@FBS-FITC/DiI-MSP@GFP fluorescent capsules to cultures of HeLa cells. The filled capsules appeared to interact with Hela cells during the first 24 h, as most nanoparticles we have previously tested in our laboratory (Figure 4b and Figure 5c). After 96 h incubation, high-resolution imaging of the cellular cytoplasm demonstrated that most intracellular capsules were located in the vicinities of the centrosome (Figure 4b). A combination of (i) filled capsules (orange color), (ii) empty capsules (green color), and (iii) capsules surrounded by the released red dye was observed throughout the cellular cytoplasm.

Serum polymerized capsules were very efficiently captured by the cells. Images taken 24 h after cell exposure to DiI-HC@FBS-FITC capsules demonstrated abundant intracellular orange (filled) fluorescent shells (Figure 5c). By 96 h incubation, a massive release of DiI, staining all the membranes of the endoplasmic reticular system, was observed in the cytoplasm of the HeLa cells. The distribution of the dye supports the hypothesis that the DiI-HC@FBS-FITC capsules were captured in endosomes where the dye is eventually released upon lysosomal enzyme activation and rupture (by enzymatic digestion) of the protein of the capsules (Figure 5c), staining the intracellular membranous structures upon lipophilic dye release.

## 3. Discussion

This work has demonstrated that MSPs can be used as sacrificial cores for the creation of PEE and protein biodegradable capsules. In the case of PEE, a multilayer coating is formed by successive adsorption of oppositely charged polymers that can be decorated with different materials, e.g., magnetic nanoparticles or carbon nanotubes, onto the charged MSP surface, following the layer-by-layer technique.

Several works have shown silica [46,47], mesoporous silica [48,49], and calcium carbonate (CaCO_3_) [50,51,52,53,54] as sacrificial cores leading to a hollow capsule. In the case of CaCO_3_ particles, their synthesis is not sufficiently reproducible producing sacrificial cores of 3 µm diameter where the intracapsular compound is incorporated in the nucleus by co-precipitation. Besides, some of these methods require the dissolution of silica by acid solutions that could damage the encapsulated compound [55]. Consequently, in addition to the important size difference (microns versus nanometers), MSP-derived hollow capsules can be filled with virtually any compound that can be included in the mesopores (i.e., under 3–4 nm diameter). Besides, MSP cores dissolve in physiological media, which preserves the integrity of all biomolecules. In addition, the PEE capsules can be fully customized by including different nanomaterials (nanoparticles or CNTs) in the PEE layers, thus improving their intrinsic properties. This would allow, for example, ‘on-demand’ compound release by applying photothermal stimuli.

More interestingly, however, in our study, we also show how to assemble polymeric capsules using completely biodegradable and biocompatible crosslinked proteins. Both, purified proteins, or complex protein mixtures—as bovine serum—can be used to produce homogeneous capsules (such is the case for GFP) or mixed protein capsules, HC@GFP and HC@FBS, respectively. As PEE capsules, these protein polymeric capsules can also be filled with a plethora of os compounds using the same simple procedure.

## 4. Materials and Methods

### 4.1. Chemicals

Poly(diallyldimethylammonium chloride) (PDDA, Mw < 100,000 (very low molecular weight), 35 wt.% in H_2_O, positively charged non-biodegradable PEE, ref. 522376), poly-L-lysine-fluorescein isothiocyanate (PLL-FITC) (Mw ~15,000–30,000) labeled, positively charged biodegradable PEE ref. 3543), poly-(sodium 4 styrenesulfonate) (PSS, Mw ~70,000, negatively charged non-biodegradable PEE, ref. 243051), and tetraethyl orthosilicate (TEOS, 98%, ref. 131903) were all purchased from Sigma-Aldrich (Madrid, Spain) and hexadecyltrimethylammonium bromide (CTAB, 99%, ref. 227160100) was purchased from ACROS Organics (The Hague, Nederlands). Multiwalled carbon nanotubes 9.5 nm in diameter and 1.5 μm in length, 95% purity, synthesized using Catalytic Chemical Vapor Deposition process, were purchased from Nanocyl (Sambreville, Belgium) as a powder, and Milli-Q water was prepared in a three-stage Millipore Milli-Q plus 185 purification system with a resistivity higher than 18.2 MΩ cm, pH 5.5.

### 4.2. Synthesis and Characterization of MSPs

MSPs were synthesized in an aqueous solution at pH 7 [56,57]. A mixture of K_2_H_2_PO_4_ (0.0504 M) and NaOH (0.029 M) in water was placed in agitation until it reached 95 °C. Then, 10 mM CTAB and 12% (*v*/*v*) glycerol were added. After the solution became homogeneous, 8.9 mM TEOS was added to the mixture every 30 min for 6 h. The mixture was further left for 2 h at 95 °C in agitation. Upon cooling, it was centrifuged at 3300× *g* for 15 min. The pellet was resuspended first, with a mixture of EtOH/H_2_O (1:1) and, later, four times more with EtOH by centrifugation/redispersion cycles (3300× *g*, 10 min). Once particles were synthesized, the CTAB was removed by thermal calcination at 600 °C for 6 h at 1 °C/min. Finally, particles were characterized by transmission electron microscopy (TEM) (for morphology and size) in a JEM 1011 (JEOL) equipped with a high-resolution Gatan digital camera. Two pore diameter sizes were quantified by adsorption isotherm of N_2_, 2.5 and 3.4 nm, and BET surface area of 346.5 m^2^/g [57].

### 4.3. MSP Coating

MSPs were coated with (i) PEE, (ii) PEE+ nanomaterials, or (iii) proteins. In the case of PEE, the sealing of the MSPs was carried out using the layer-by-layer technique [58]. For the coating, MSPs were incubated in a 0.25 mg/mL solution of PLL-FITC or PDDA in 0.5 M NaCl. The mixture was kept under stirring for 30 min at room temperature. The excess of PEE was washed by three centrifugation/resuspension cycles in water (3300× *g*, 20 min). The number of layers can be incremented (depending on the resistance wanted for the capsule) using alternating positive and negative PEE following the described protocol. MSPs can also be precoated with PEE (positive, i.e., PLL) and covered with a nanomaterial. To protect the structure, a mesoporous silica layer was formed over the magnetic hybrid. In the other example, MSPs were coated with oxidized multi-walled carbon nanotubes (CNTs). These were pre-treated with acetone and ethanol to remove organic materials, subsequently, they were oxidized in a mixture of H_2_SO_4_/HNO_3_ (3:1); a stable dispersion (1.54 mg/mL) of oxidized CNTs holding a negative surface charge was obtained, as previously described [32]. The CNTs were attached to previously sealed MSPs coated with a positively charged PEE following the procedure previously described [59]. Excess CNTs were removed by centrifugation/redispersion cycles in water (3300× *g*, 20 min). Alternatively, MSPs were also coated with synthesized CoFe_2_O_4_ nanoparticles, deposited on top of the PEE layer, as described elsewhere [60]. MSPs were coated with successive PEE layers and 9 mg of CoFe_2_O_4_ was added to the negatively charged MSPs solution. MSP@CoFe_2_O_4_ were coated with an extra mesoporous silica layer by adding 156.25 mg of CTAB, 62.5 mL of H_2_O, 25 mL of EtOH, 0.75 mL of NH_4_OH, and 1.03 mL of TEOS. MSPs were also coated with dendritic Pt nanoparticles; they were deposited on top of the subsequent polyelectrolyte layers over the silica. These nanoparticles were synthesized as previously described elsewhere [61]. Then, 5 mg of MSP@Pt were covered with 120 µL of CNTs (0.64 mg/mL) after three polyelectrolyte deposition layers (MSP@NPs@CNTs).

Regarding proteins, MSPs were incubated either with 30% fetal bovine serum (FBS) in Dulbecco’s modified Eagle medium (DMEM) or in PBS for purified fluorescent protein (6xHis:green fluorescent protein (GFP)). FBS was incubated for 12 h in mild rotation. The 6xHis:GFP protein coating was made following the procedure previously described [43]. The excess of protein was washed by three centrifugation/resuspension cycles (3300× *g* for 10 min) in water. Cross-linking of the protein coating was performed with glutaraldehyde (Sigma-Aldrich ref. G6257) at 0.25% for 20 min. Glutaraldehyde creates stable secondary amine linkages producing a stable protein mesh. Excess glutaraldehyde was repeatedly washed using centrifugation/resuspension cycles (3300× *g* for 10 min).

### 4.4. MSPs Loading

To track the particles and determine the burst-release point of the capsule *in cellulo*, MSPs were loaded with a lipophilic dye called DiI (1,1′-dioctadecyl-3,3,3′,3′-tetramethylindocarbocyanine perchlorate, Merk Ref. 468495). For the encapsulation, 0.5 mg of MSPs was incubated in a solution containing 0.32 mg/mL of DiI in 5% dimethyl Sulfoxide (DMSO)/water for 3 h at room temperature (RT) in mild agitation. Excess of dye was first washed with 5% DMSO and later, with centrifugation/redispersion cycles in the water at 3300× *g* for 10 min.

### 4.5. Dissolution of the Silica Core

Here, 100 µg/mL of MSPs was incubated in a buffered salt solution (BSS, PANBiotech) for different times at 37 °C in agitation or as described in the text of the manuscript. BSS composition (400 mg/L KCl, 8.000 mg/L NaCl, 47.88 mg/L Na_2_HPO_4_, 60 mg/L KH_2_PO_4_, 1.000 mg/L C_6_H_12_O_6_, and 350 mg/L NaHCO_3_) is similar to phosphate-buffered saline (PBS) in terms of salt composition/pH, but was chosen as it is a commonly used as media for *in cellulo* testing. Particle core dissolution was confirmed using TEM.

### 4.6. Cell Culture, Fluorescent Cell Labeling, Confocal Microscopy, and Viability

HeLa cells were obtained from the European Molecular Biology Laboratory Cell Bank. Cells were cultured under standard conditions in Eagle’s minimal essential medium (MEM) containing 10% FBS and antibiotics (from Gibco, Thermo Fisher Scientific, Waltham, MA, USA). Cells were incubated with the equivalent to 10 µg/mL of particles (prior to silica removal). The hollow capsules were resuspended and functionalized by mild sonication in standard tissue culture medium containing serum. For imaging purposes, cells were fixed in 4% paraformaldehyde. Nuclei were stained with Hoechst dye (Bisbenzimide, Sigma-Aldrich Ref. B2883). Confocal microscopy images were obtained with a Nikon A1R confocal microscope and were processed with the NIS-Elements Advanced Research software. High-resolution confocal imaging was performed using a Nikon Plan Apochromatic 100× oil numerical aperture 1.45 objective. All confocal cell images are pseudocolored.

## 5. Conclusions

One of the problems of nanomedicine is to precisely control the side effects (degradation, accumulation) of nanomaterials used as platforms for drug delivery in vivo. In this sense, the polymeric capsules described in this manuscript represent a breakthrough. They are fully biocompatible structures that can be assembled by cross-linking many different types of biologically compatible and easily biodegradable polymers, with little or no toxicity, such as proteins. This versatility allows this new encapsulation system to open new lines for the engineering of fully customizable encapsulation systems, for example, to create capsules of fluorescent proteins or ligand proteins, which could be an effective strategy to create targeted capsules. Besides, these capsules can be customized with different nanomaterials depending on the desired final application, thus offering wide application possibilities, ranging from imaging to drug administration.
